# An updated definition of V(D)J recombination signal sequences revealed by high-throughput recombination assays

**DOI:** 10.1093/nar/gkac1038

**Published:** 2022-11-12

**Authors:** Walker Hoolehan, Justin C Harris, Jennifer N Byrum, Destiny A Simpson, Karla K Rodgers

**Affiliations:** Department of Biochemistry and Molecular Biology, University of Oklahoma Health Sciences Center, Oklahoma City, OK 73104, USA; Department of Biochemistry and Molecular Biology, University of Oklahoma Health Sciences Center, Oklahoma City, OK 73104, USA; Department of Biochemistry and Molecular Biology, University of Oklahoma Health Sciences Center, Oklahoma City, OK 73104, USA; Department of Biochemistry and Molecular Biology, University of Oklahoma Health Sciences Center, Oklahoma City, OK 73104, USA; Department of Biochemistry and Molecular Biology, University of Oklahoma Health Sciences Center, Oklahoma City, OK 73104, USA

## Abstract

In the adaptive immune system, V(D)J recombination initiates the production of a diverse antigen receptor repertoire in developing B and T cells. Recombination activating proteins, RAG1 and RAG2 (RAG1/2), catalyze V(D)J recombination by cleaving adjacent to recombination signal sequences (RSSs) that flank antigen receptor gene segments. Previous studies defined the consensus RSS as containing conserved heptamer and nonamer sequences separated by a less conserved 12 or 23 base-pair spacer sequence. However, many RSSs deviate from the consensus sequence. Here, we developed a cell-based, massively parallel assay to evaluate V(D)J recombination activity on thousands of RSSs where the 12-RSS heptamer and adjoining spacer region contained randomized sequences. While the consensus heptamer sequence (CACAGTG) was marginally preferred, V(D)J recombination was highly active on a wide range of non-consensus sequences. Select purine/pyrimidine motifs that may accommodate heptamer unwinding in the RAG1/2 active site were generally preferred. In addition, while different coding flanks and nonamer sequences affected recombination efficiency, the relative dependency on the purine/pyrimidine motifs in the RSS heptamer remained unchanged. Our results suggest RAG1/2 specificity for RSS heptamers is primarily dictated by DNA structural features dependent on purine/pyrimidine pattern, and to a lesser extent, RAG:RSS base-specific interactions.

## INTRODUCTION

Functional immunoglobulin and T cell receptor (TCR) genes are generated in developing B and T cells, respectively, through multiple V(D)J recombination reactions (1,2). This process leads to a diverse repertoire of antigen receptors (AgR) in the adaptive immune system, which are capable of recognizing a vast array of foreign antigens. V(D)J recombination joins V, D and J gene (coding) segments to construct AgR genes through a cut-and-paste mechanism. V(D)J recombinase specificity guides AgR gene assembly by dictating gene segment utilization in developing B- and T-cells. Gene segments within the same class, such as V_H_ gene segments, are not equally utilized during V(D)J recombination, resulting in biased AgR expression (3).

The recombination activating proteins, RAG1 and RAG2 (RAG1/2), facilitate recombination by recognizing recombination signal sequences (RSSs) that flank the coding segments (4). Each RSS contains a relatively conserved heptamer and nonamer sequence separated by a poorly conserved 12 or 23 base-pair (bp) spacer sequence, known as the 12-RSS and 23-RSS, respectively. To stimulate recombination, a paired complex (PC) is formed consisting of a RAG1/2 heterotetramer bound to both a 12-RSS and a 23-RSS, in accordance with the ‘12/23 rule’ (5,6). Upon PC formation, RAG1/2 cleaves DNA at RSS/coding junctions in a nick and hairpin-forming transesterification reaction, resulting in hairpin-sealed coding ends and open double-strand breaks at the 5′ ends of each RSS, termed signal ends (7). RAG1/2 releases the coding ends to nonhomologous end joining (NHEJ) factors and remains stably bound to the signal ends in a signal end complex (SEC) (8–10). The hairpin coding ends are opened and processed by addition or deletion of bases followed by coding end joining, which produces the exons encoding the variable region of the AgR. Signal ends are later joined heptamer-to-heptamer, typically with a precise junction, to form a signal joint (SJ).

In addition to shaping the AgR repertoire, V(D)J recombinase specificity has important implications for genomic stability. Previous studies showed RAG1/2 can cleave non-consensus RSSs (11–17). Off-target RAG-mediated cleavage events involving cryptic RSSs occur in a large subset of RAG-expressing pre-B-cells (14). Subsequent aberrant joining of the cleaved cryptic RSSs can result in chromosomal abnormalities and oncogenesis (18,19). Numerous co-factors can promote or inhibit V(D)J recombination, but recent studies have highlighted the importance of RAG1/2 DNA sequence specificity (20,21). A consensus RSS, CACAGTG-N_12/23_-ACAAAAACC, was derived from conservation of annotated RSSs (22). However, many RSSs in AgR loci diverge from the consensus RSS; therefore, RAG1/2 must be promiscuous enough to cleave divergent RSSs and generate AgR diversity, but it must also be precise to avoid cleaving cryptic RSSs and cause genomic instability.

We developed the selective amplification of recombination products and sequencing (SARP-seq) method to evaluate DNA selectivity in the V(D)J recombination reaction in an unbiased and high throughput approach. From the SARP-seq results described here, we provide the most comprehensive characterization of V(D)J recombinase specificity for RSSs to date. In a massively parallel format, these findings demonstrate RAG1/2 preference for selected purine/pyrimidine motifs in the RSS heptamer, elucidate effects of flanking heptamer sequences and the nonamer on recombination activity, and reveal DNA sequence-specific features that may enhance RAG-mediated cleavage through molecular dynamics simulations. Further, we demonstrate how SARP-seq results can be used to elucidate the relative contribution of RSS sequence to gene utilization at an endogenous AgR locus.

## MATERIALS AND METHODS

### pMX-INV and pMAX-INV extrachromosomal recombination substrates

The extrachromosomal recombination substrates, pMX-INV (kindly provided by Barry Sleckman) and pMAX-INV (generated as described below), contain a consensus 12-RSS and a consensus 23-RSS in a direct repeat orientation (plasmid maps shown in Figure S1). Classical extrachromosomal V(D)J recombination assays using either pMX-INV or pMAX-INV results in an inversional recombination product in the presence, but not the absence, of RAG1/2 (example with pMX-INV as substrate is shown in Supplementary Figure S2A). pMAX-INV was created by NEBuilder assembly. Parent construct pmaxGFP (Lonza Bioscience) was amplified with two primer pairs: ‘Vec FWD’ + ‘Vec RVS’ and ‘Insrt FWD’ + ‘Insrt RVS’ (Supplementary Table S1). PCR products were purified with a Zymo DCC-5 kit. Purified PCR products were assembled with NEBuilder® HiFi DNA Assembly Master Mix (NEB # E2621S) following manufacturer instructions. Assembled pMAX-INV was transformed into DH5α cells (ThermoFisher Scientific # 18258012) and isolated by miniprep (Qiagen # 27104). The miniprepped pMAX-INV was sequenced to confirm accurate assembly by targeted Sanger sequencing (Sanger DNA sequencing facility, Oklahoma Medical Research Foundation) and by whole plasmid sequencing (plasmidsaurus).

### Input library preparation

To generate the SARP-seq input plasmid substrate library, pMX-INV or pMAX-INV was digested with 5 units of EcoRI-HF (NEB # R3101S) per μg of DNA followed by digestion with 5 units of MluI-HF (NEB # R3198S) per μg of DNA. Digested plasmid was purified by extraction from a 1% agarose gel using the Monarch DNA Gel Extraction Kit following manufacturer instructions (NEB # T1020S). Partially degenerate DNA oligonucleotide (sequences in Supplementary Table S1) was duplexed with Duplexing Primer (sequence in Supplementary Table S1) by a single annealing and primer extension step with Q5 high-fidelity polymerase. Duplexed DNA was digested with 4 units of EcoRI-HF per 250 ng of DNA followed by digestion with 4 units of MluI-HF per 250 ng of DNA, and subsequently purified using the Zymo DNA Clean and Concentrator (DCC) Kit (Zymo # D4013). The double-digested and gel purified plasmid and duplexed oligo were ligated at a 3:1 oligo:plasmid ratio with T4 DNA Ligase by overnight incubation at 16°C and purified using the Zymo DCC kit. The resulting ligation product was used as an input library for extrachromosomal V(D)J recombination experiments in the SARP-seq protocol. In summary, input library preparation was used to construct three separate libraries: pSARP-12R4-9, pSARP-cf12R4-7 and pSARP-MAX-cNON (Supplementary Table S2).

### Extrachromosomal V(D)J recombination experiment and SARP-seq

HEK293T cells were seeded in a 10 cm plate and grown overnight to 55–75% confluency in media containing DMEM, 10% FBS, 1× antibiotic-antimycotic and 1 mM sodium pyruvate. The cells were then co-transfected with an expression vector for maltose binding protein core-RAG1 fusion protein (MBP-cRAG1) (kindly provided by Patrick Swanson), an expression vector for mCherry-tagged core-RAG2 (Ch-cRAG2) (23), and the pSARP-12R4-9 or pSARP-cf12R4-7 input library with a 3:1 transfection reagent:DNA ratio using Fugene 6 transfection reagent (Promega #E269A). The pSARP-MAX-cNON input library was co-transfected with an expression vector for mCherry-tagged core-RAG1 (Ch-cRAG1) in lieu of MBP-cRAG1. At 72 h, ∼90% of cells were transfected, as as visualized by fluorescence microscopy for the proportion of cells expressing mCherry. Plasmid DNA was then purified and signal joints present in the recovered plasmid were selectively amplified with a nested PCR approach as specified for each SARP-seq library in Supplementary Figure S3 and Table S2. The PCR amplicon was PAGE purified, and primers indicated in Supplementary Table S1 and Figure S3 used to amplify output libraries. The final PCR products were PAGE purified and subjected to Illumina next generation sequencing on the iSeq 100 or miSeq platform (iSeq runs were performed at the OUHSC Nathan Shock Center of Excellence in the Biology of Aging, and the miSeq run was performed at the OUHSC Laboratory of Molecular Biology and Cytometry Research). ISeq libraries were loaded at a 50 pM concentration with 50% PhiX spike-in, and the miSeq library was loaded at an 8 pM loading concentration with a 50% PhiX spike-in.

### Analysis of next-generation sequencing data

Galaxy workflows for analyzing SARP-seq data are provided as supplementary data and at the following URL: https://github.com/RodgersLab/SARP-seq.git. The workflow was slightly modified to account for variations in experimental design, including sample-specific index matching to separate coding flank variants. In brief, reads containing the precise signal joint sequence ‘GTGCACAGTG’ were matched, and matching reads with mean *Q* < 20 were filtered out with PRINSEQ (24). Precise signal joints served as an index for sequences of interest and a positive control for RAG-mediated complete V(D)J recombination. Next, all bases except the 12-RSS heptamer and the first two nucleotides of the 12-RSS spacer were trimmed, and reads containing any base with *Q* < 20 were filtered out. Reads were converted to FASTA format and matched for ‘CAC’ in the first three positions of the RSS heptamer. Unique sequence read occurrences were counted.

### Molecular dynamics simulations

Initial models for molecular dynamics simulations were generated by modeling recombination signal sequences into a B-DNA structure using the ‘fiber’ command within X3DNAv2.4 (25). B-DNA was simulated using the parmbsc1 force-field implemented in GROMACS-2019 (26,27). The RSS models were placed in a solvent box containing a 10 nm buffered layer of explicit tip3p water molecules. The solvent box was neutralized by randomly replacing solvent water molecules with potassium ions. The system was subject to energy minimization using <50 000 iterations of a steepest descent algorithm or until the system force < 1000 kJ/mol/nm. The minimized structure was used as an input for 100 ps temperature equilibration to 300 K using a weak-coupling modified Berendsen thermostat. The temperature equilibrated system was used as an input for 100 ps of pressure equilibration to 1 bar using an isotropic Parrinello-Rahman barostat. After pressure equilibration, the system was subject to 100 ns of production simulation time. Simulations were run with a leapfrog integrator over a 2 fs time step. Hydrogen bonds were constrained by fourth order lincs algorithm. Nonbonded interactions were calculated using a Verlet cutoff scheme with a 1 nm cutoff for both short-range electrostatic interactions and short-range Van der Waals interactions. Particle Mesh Ewald summation with 0.15 nm grid spacing was used to calculate long-range electrostatic interactions.

### Analysis of molecular dynamics data

Post-production processing and analysis of molecular dynamics simulation data was performed using GROMACS-2019 (https://doi.org/10.5281/zenodo.3685922) and X3DNA software packages (25,26). Molecules of interest were centered in the solvent box, and trajectory files were converted to PDB format. The first 20 ns of simulation time were excluded from further analysis to allow for equilibration. A PDB file containing coordinates for each simulated time-step was used as an input for X3DNA’s ‘analyze’ program, which generated a complete, reversible set of helical and base-pair step parameters capable of rebuilding the DNA molecules of interest (25). Base-pair step parameters and minor groove widths for each recorded time-step were used to generate molecular dynamic figures. Minor groove widths were calculated as a simple inter-phosphate distance using the X3DNA software package (25,28).

### PAGE purification

The PCR product was separated on a 10% polyacrylamide gel for ∼1 h at 125 V with a cooling circulating water bath. The gel was stained with 1x SYBR safe (Thermofisher #S33102), visualized under blue light, and the band of interest was excised from the gel. The PCR product was eluted from the gel slice by crushing the gel slice and soaking in TE buffer (pH 7.5) buffer overnight (‘crush-and-soak’ method). The PCR product was further purified using a Zymo DNA Clean and Concentrator Kit (Zymo # D4013).

### Low throughput extrachromosomal V(D)J recombination assay

The 12-RSS in pMX-INV and pMAX-INV were replaced with a consensus (CACAGTG-AT) or anti-consensus (CACGTAC-AT) 12-RSS. For experiments performed in HEK293T cells, the pMX-INV constructs were separately transfected into HEK 293T cells with the MBP-core-RAG1 and mCherry-core-RAG2 expression vectors, and after 72 hrs, the plasmid DNA was purified as in the SARP-seq protocol. For experiments performed in pre-B-cells, 5 μg of each pMAX-INV construct was separately electroporated into v-abl pre-B cells (A70.2 cell line provided by Barry Sleckman). Electroporation was performed on a Bio-Rad Gene Pulser II at 950 μF using a cuvette with a 0.4 cm gap width. 72 h after electroporation, A70.2 cells were treated with 3 μM STI-571 to arrest in G_1_-phase, inducing expression of endogenous RAG1/2 (29). Cells were harvested after 96 h of STI-571 treatment, and total DNA was purified by overnight 37°C incubation in digestion buffer (see *extrachromosomal V(D)J recombination experiment and SARP-seq*) with shaking and subsequent ethanol precipitation. For experiments performed in both HEK293T and A70.2 cells, purified DNA was serially diluted by 2-fold two times and both dilutions, along with undiluted purified DNA, were subjected to PCR amplification of V(D)J recombined DNA, and undiluted total purified DNA was PCR amplified as an input control. Total pMX-INV DNA was amplified using primers ‘Nest FWD’ and ‘Input RVS’ and V(D)J recombined pMX-INV was amplified using primers ‘Nest FWD’ and ‘Nest RVS’ (Supplementary Table S1). Total purified pMAX-INV DNA was amplified with primers ‘MAX FWD’ and ‘MAX input,’ and V(D)J recombined plasmid DNA was amplified with primers ‘MAX FWD’ and ‘MAX RVS.’ pMX-INV PCR products were separated on an 8% polyacrylamide gel, stained with SYBR safe, and imaged. pMAX-INV PCR products were separated on a 1% agarose gel, stained with SYBR safe, and imaged. Band intensities were quantified, and recombination activity calculated by dividing recombined DNA band intensity by input DNA band intensity. Experiments were repeated 3 times (*n* = 3) and data were normalized to 1 for the consensus RSS (Supplementary Figure S5E and F).

### Statistics

Statistical tests were performed in GraphPad Prism 9 and are described in figure legends where applicable. When two groups were compared, a two-tailed Student's *t*-test was performed. When more than two groups were compared, an ordinary one-way ANOVA with Dunnett's multiple comparisons test was performed. Asterisks denote *P*-value range, where *P < 0.05; **P < 0.01; ***P < 0.001 and ****P < 0.0001.

## RESULTS

### Selective amplification of recombination products and sequencing (SARP-seq)

**Figure 1.**
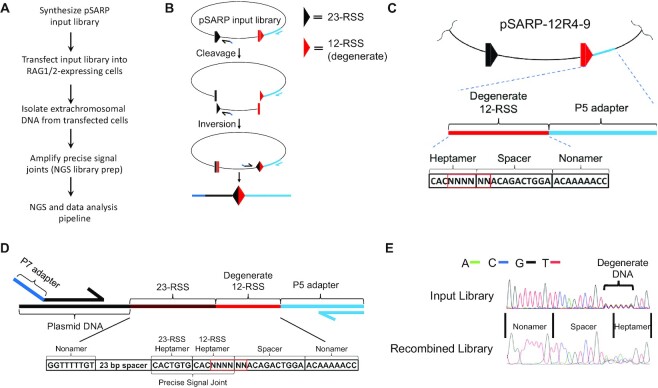
Schematic overview of SARP-seq method. (**A**) Flow chart of SARP-seq protocol. (**B**) Diagram of episomal V(D)J recombination. The 12- and 23-RSSs are represented as red and black triangles, respectively. Half arrows represent PCR primers used to selectively amplify recombination products. The P5 adapter (light blue) and P7 adapter (dark blue) sequences are listed in *Supplementary Table S1*. Coupled cleavage of 12- and 23-RSSs is followed by coding end (rectangles) joining and signal end joining by NHEJ. Joining of coding and signal ends results in an inversional recombination event. (**C**) Diagram of partially degenerate 12-RSS in pSARP-12R4-9 input library flanked by P5 adapter sequence. The non-degenerate spacer sequence is from murine VκL8 12-RSS, shown previously to impart good recombination efficiency (17). (**D**) Diagram of final PCR amplicon of the recombined pSARP-12R4-9 that was subjected to NGS. (**E**) Electropherogram charts of 12-RSS in pSARP-12R4-9 input library and of V(D)J recombination products (recombined library), showing the reverse complement sequence of the 12-RSS.

The SARP-seq method builds on a well-established approach that assays V(D)J recombination activity in RAG1/2-expressing cells using extrachromosomal plasmid substrates (29,30). In the standard assay, cells transfected with substrate plasmids are cultured for several days, followed by recovery of plasmid. Recombined plasmid products are detectable from cells expressing RAG1/2, but absent in cells expressing only one or neither of the RAG proteins (example shown in Supplementary Figure S2A). Here, we coupled the extrachromosomal recombination assay with next-generation sequencing (NGS) to simultaneously determine the relative utilization of thousands of RSSs in a complete V(D)J recombination reaction (Figure 1A–E). This was accomplished by randomizing the DNA sequence within an RSS to yield a large library of plasmid substrates (31). The utilization of each RSS in the recombination reaction was subsequently determined by NGS and a custom data analysis pipeline (Supplementary Figure S4).

First, SARP-seq was used to determine selectivity for the 3′ portion of the 12-RSS heptamer and adjacent spacer region in V(D)J recombination. This region is only semi-conserved amongst endogenous RSSs. The RAG complex induces this region of the RSS to undergo a series of conformational changes during DNA cleavage, and yet the sequence specificity that facilitates this process is not well-defined. To generate the plasmid substrate input library, pSARP-12R4-9, a 12-RSS containing randomized sequences through the heptamer and neighboring spacer was paired with a consensus 23-RSS in the pMX-INV vector backbone (Figure 1C–E). The first three positions of the heptamer remained constant in the input library, as the CAC sequence is required for RAG-mediated DNA cleavage (32). The next six consecutive base-pairs, including 4 bp at the 3′-end of the heptamer plus the first 2 bp of the spacer, were fully randomized with equal ratios of A:C:G:T at each position. Full degeneracy of positions 4–9 in the resulting pSARP-12R4-9 input library was confirmed by Sanger DNA sequencing (Figure 1E).

V(D)J recombination of the pSARP-12R4-9 library leads to inversional rearrangement with the resulting signal and coding joints retained in the plasmid (Figure 1B). To initiate V(D)J recombination activity, the pSARP-12R4-9 library and core RAG1 and RAG2 expression vectors (MBP-cRAG1 and Ch-cRAG2) were co-transfected into 293T cells. Plasmid DNA was isolated 72 h after co-transfection, allowing sufficient time for extrachromosomal V(D)J recombination (Supplementary Figure S2A). The recombined output library was generated by amplifying RSS signal joints (Figure 1B, D and E). Sanger sequencing showed partial loss of degeneracy of the 12-RSS consistent with RAG1/2 selection of certain RSSs in V(D)J recombination (Figure 1E). The output library was then subjected to NGS, and read counts for RSSs that formed precise signal joints were tabulated. In a negative control experiment, no loss of sequence degeneracy was observed for output library isolated from transfected 293T cells that was not co-transfected with RAG1/2 expression vectors (Supplementary Figure S2C and D).

**Figure 2.**
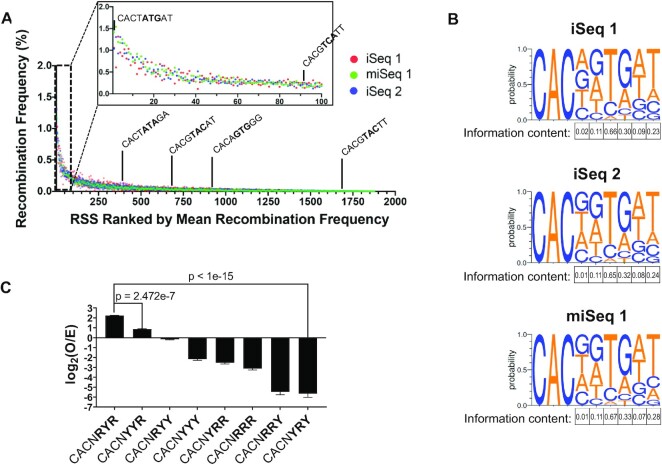
DNA sequence selectivity determined by SARP-seq. SARP-seq was replicated 3 times on two different sequencing platforms: twice on the iSeq 100 (iSeq 1 and iSeq 2) and once on the miSeq (miSeq 1). (**A**) Recombination frequencies of individual RSSs expressed as a percentage of total recombination events. Every RSS with reproducible V(D)J recombination activity was ranked by mean recombination frequency, so more efficacious RSSs occupied higher ranks and less efficacious RSSs occupied lower ranks. Efficacy was determined by calculating the mean recombination frequency of all three replicates (Supplementary Dataset S1). Specific RSSs are indicated by black pointers and listed from most efficacious to least efficacious: The top-ranked consensus R/Y motif (CACTATGAT), top-ranked RSS that completely lacks canonical consensus RSS base identity for heptamer positions 4–7 (CACGTCATT), median-ranked consensus R/Y motif (CACTATAGA, top-ranked anti-consensus R/Y motif (CACGTACAT), bottom-ranked canonical consensus RSS (CACAGTGGG), and median ranked anti-consensus R/Y motif (CACGTACTT). The top 100 RSSs are magnified in the panel inset. (**B**) Sequence logos depicting RAG1/2 specificity expressed as the probability of finding each base in precise signal joints. Total information content for each position of the degenerate 12-RSS region was calculated and expressed in nats. (**C**) Bar chart showing V(D)J recombination frequency of different purine/pyrimidine sequence motifs. Recombination frequencies are expressed as log_2_(O/E). *O* is the observed frequency of recombination events, and *E* is the expected frequency of random, non-specific recombination. Positive values indicate positive selection, and negative values indicate negative selection. Statistically significant differences were determined by ordinary one-way ANOVA with Dunnett's multiple comparisons test (*n* = 3) (****P < 0.0001).

SARP-seq experiments using the pSARP-12R4-9 input library were replicated 3 times, and the resulting libraries were sequenced twice on the iSeq 100 platform and once on the miSeq platform. We refer to our first and second iSeq runs as ‘iSeq 1’ and ‘iSeq 2’, respectively, and the miSeq run is referred to as ‘miSeq 1’. For iSeq 1 and iSeq 2, the entire SARP-seq protocol was repeated starting with preparation of the pSARP-12R4-9 input library, and miSeq 1 utilized the same degenerate 12-RSS input library as iSeq 2. The SARP-seq output libraries were sequenced at sequencing depths varying by >2 orders of magnitude. 12-RSSs utilized during V(D)J recombination were analyzed using a custom data analysis pipeline (Supplementary Figure S4 and described in Materials and Methods). In brief, reads containing a precise signal joint were extracted from FASTQ files, as precise signal joints are a hallmark of V(D)J recombination (Figure 1D). Differential read counts of precisely joined 12- and 23-RSSs correspond to V(D)J recombination frequencies. 12-RSS logos generated from precise signal joint read counts show striking reproducibility (Figure 2B). Information content for each position of the RSS heptamer was highly reproducible, and read counts for specific RSS motifs were reproducible across each independent SARP-seq experiment (Figure 2A and B).

Analysis of the SARP-seq results revealed a clear hierarchy of 12-RSSs utilized in V(D)J recombination (Figure 2A and C, and Supplementary Figure S5). For example, >1800 unique 12-RSSs were found to reproducibly form a precise signal joint with the canonical 23-RSS, and the top 20 sequences made up 16.7% of all precise signal joints (Dataset S1). Conversely, >1600 unique sequences were poorer substrates that each made up <0.1% of total precise signal joints. Those sequences accounted for 40.1% of all precise signal joints despite accounting for 87.4% of unique sequences that reproducibly form precise signal joints (Dataset S1).

### The R/Y pattern in the RSS heptamer is highly predictive of RAG1/2 activity

By including all CAC-containing heptamers, SARP-seq deciphered heptamer features that increase V(D)J recombination. Five unique heptamer sequences encompassed 5.7% of total reads for precise signal joints (Supplementary Dataset S1). Four of these sequences differed only in position 4 of the CACNGTG motif. Interestingly, in addition to CACNGTG, the CACTATG heptamer was consistently in the top 2–3 heptamers used (Figure 2A and Supplementary Dataset S1). CACTATG is the heptamer sequence in the terminal inverted repeat (TIR) of the ProtoRag transposon identified from *Branchiostoma belcheri* (Bb) (33). While mammalian RAG enzymes efficiently cleave RSS substrates containing the Bb RSS heptamer, this heptamer sequence is poorly represented in endogenous RSSs at AgR loci in both mice and human (34).

The sequence logos from each experiment represent the base preference at each position (Figure 2B). Based on the sequence logo, position 4 in the RSS heptamer showed the least discrimination of base identity amongst all the heptamer base positions (Figure 2B). The preference for heptamer positions 5–7 largely depended on the purine (R) or pyrimidine (Y) classification of each base (Figure 2B and C). For example, R (A or G) was preferred over Y (C or T) at positions 5 and 7 by approximately 2-fold and 7-fold, respectively (Figure 2B and C). Notably, the R/Y specificity was most pronounced at position 6, where Y over R was preferred by greater than one order of magnitude (Figure 2B and C). Consistent with the selection against purines at this position, SARP-seq results show > 80% of RSS heptamers that contained G at position 6 were in the bottom fourth of RSSs utilized in the inversional V(D)J recombination reactions (Figure 2B and Supplementary Dataset S1).

Normalized recombination frequencies for every RSS studied were ranked by their mean recombination frequency with the more efficacious RSSs ranked higher than less efficacious RSSs (Figure 2A). The top-ranked and median-ranked consensus R/Y motifs (CACNRYR) were ranked #1 and #400, respectively. In contrast, the top-ranked and median-ranked anti-consensus R/Y motifs (CACNYRY) were ranked #694 and #1652, respectively. While the consensus R/Y motif is a feature shared with the canonical consensus RSS (CACAGTG), the consensus R/Y motif provides a much more accurate, comprehensive description of V(D)J recombination specificity. The canonical consensus RSS describes less than 5% of V(D)J recombination products, and the canonical consensus heptamer is only the 933rd most efficacious RSS when flanked by a ‘GG’ spacer sequence (Figure 2A and Dataset S1). Interestingly, the sequence ‘CACGTCATT’ shares no base identity with canonical consensus RSS at heptamer positions H4-H7 but is a top-100 12-RSS, vastly outcompeting the canonical consensus RSS sequence ‘CACAGTGGG’ (Figure 2A). The consensus R/Y sequence, however, describes more than 50% of all V(D)J recombination products (Figure 2C). We quantified RSS utilization for every R/Y motif spanning positions 5–7 (Figure 2C). R/Y specificity is striking, with CACNRYR preferred > 100-fold more than CACNYRY (Figure 2C). Nevertheless, the ‘consensus’ R/Y motif CACNRYR was only enriched by ∼4-fold relative to simulated random RSS selection (Figure 2C). Most R/Y motifs were depleted by > 4-fold compared to random sequence selectivity, with > 16-fold depletion for CACNRRY and CACNYRY (Figure 2C). Thus, while a specific consensus motif is not strongly selected for V(D)J recombination, some R/Y motifs are strongly selected against (Figure 2C). We also quantified V(D)J recombination activity for every possible combination of ‘weak’ A/T (W) and ‘strong’ G/C (S) sequences (Supplementary Figure S5D). CACNSWS was favored by <10-fold over the least active S/W motif, CACNSSW, demonstrating that the R/Y pattern is a more accurate representation of RSS sequence motif preference for V(D)J recombination. To further validate the preference for CACNRYR over CACNYRY, we performed low-throughput extrachromosomal V(D)J recombination assays by cloning an active consensus (CACAGTGAT) and anti-consensus (CACGTACAT) RSS into pMX-INV and performing semi-quantitative V(D)J recombination assays (*Materials and Methods* and Supplementary Figure S5E). A statistically significant difference in V(D)J recombination activity was observed for the same consensus and anti-consensus RSSs in both the low-throughput assay and SARP-seq (Supplementary Figure S5E). Further, this divergence in recombination efficiencies between the consensus and anti-consensus 12-RSS heptamers was also observed in pre-B cells endogenously expressing RAG1 and RAG2 (Supplementary Figure S5F).

### The heptamer-flanking spacer sequence is a major determinant for RSS utilization

While the 12 and 23 bp spacers are the least conserved regions of the RSS, previous studies showed some DNA sequence preferences at the 5′ end of the spacer in the 12-RSS (17,35). In the pSARP-12R4-9 input library, the first two positions of the 12 bp spacer were fully randomized, such that each RSS heptamer is sampled with 16 different spacer sequences. The SARP-seq results can be summarized in two main points. First, the relative ranking of the 16 spacers show that the top 4 ranking spacers all include T at the second position (position 9 in the 12-RSS), and a preference for A or T at the first position of the spacer (Table 1 and Supplementary Figure S6A and B). The poorest spacers are G/C rich, with CG and GG as the bottom two ranked spacer sequences (Table 1 and Supplementary Figure S6A and B). Second, the adjoining spacer sequence has profound effects on heptamer utilization (Table 1 and Supplementary Figure S6C–F). For example, the frequency at which the consensus CACAGTG heptamer is utilized varies considerably depending on the adjoining spacer sequence, with a >50-fold difference in observed/expected values for the CACAGTG consensus heptamer flanked by a ‘good’ versus a ‘poor’ spacer (Figure 2A and Dataset S1). The frequency with a ‘poor’ spacer brings the utilization of the CACAGTG heptamer to similarly low levels as sequences containing anti-consensus CACNYRY or CACNRRY heptamer motifs (Figure 2A).

**Table 1.** Top RSS heptamer sequences for each spacer sequence falling in the top quartile (upper tables) or bottom quartile (lower tables) of recombination frequency. Count frequency data taken from Supplementary Dataset S1

**AT spacer**		**TT spacer**		**GT spacer**		**CT spacer**	
CACTATGAT	1.168	CACTGTGTT	1.141	CACTATGGT	0.516	CACAGTGCT	1.150
CACAGTGAT	1.144	CACGGTGTT	0.925	CACAGTGGT	0.452	CACTATGCT	0.557
CACTGTGAT	1.093	CACAGTGTT	0.829	CACTGTGGT	0.448	CACTGTGCT	0.515
CACGGTGAT	1.036	CACTATGTT	0.805	CACGGTGGT	0.344	CACGGTGCT	0.499
CACCGTGAT	0.952	CACCGTGTT	0.721	CACAATGGT	0.344	CACCGTGCT	0.442
CACCATGAT	0.755	CACGTTGTT	0.620	CACGATGGT	0.338	CACAATGCT	0.330
CACGATGAT	0.739	CACAATGTT	0.583	CACCATGGT	0.328	CACCATGCT	0.317
CACAATGAT	0.726	CACGATGTT	0.516	CACCGTGGT	0.313	CACGTTGCT	0.247
CACGTTGAT	0.680	CACATTGTT	0.508	CACGTTGGT	0.243	CACGATGCT	0.228
CACATTGAT	0.597	CACCATGTT	0.504	CACTGTAGT	0.242	CACTATACT	0.157
**GG spacer**		**CG spacer**		**CC spacer**		**GA spacer**	
CACGTTGGG	0.066	CACTGTGCG	0.103	CACCGTGCC	0.131	CACTATGGA	0.106
CACTATGGG	0.064	CACGGTGCG	0.088	CACAGTGCC	0.128	CACTGTGGA	0.104
CACGATGGG	0.054	CACCGTGCG	0.085	CACTGTGCC	0.128	CACGGTGGA	0.084
CACCGTGGG	0.040	CACGATGCG	0.073	CACTATGCC	0.119	CACGGTAGA	0.075
CACTGTGGG	0.037	CACTATGCG	0.072	CACGATGCC	0.109	CACCGTGGA	0.066
CACCATGGG	0.031	CACAGTGCG	0.052	CACGGTGCC	0.090	CACTATAGA	0.062
CACAATGGG	0.029	CACATTGCG	0.041	CACAATGCC	0.089	CACAGTGGA	0.060
CACGGTAGG	0.027	CACCATGCG	0.040	CACCATGCC	0.072	CACCATGGA	0.054
CACGGTGGG	0.022	CACAATGCG	0.036	CACGTTGCC	0.050	CACGTTGGA	0.053
CACAGTCGG	0.021	CACGTTGCG	0.033	CACATTGCC	0.049	CACAGTAGA	0.051
CACAGTGGG	0.020						

*Canonical RSS heptamer sequences are highlighted.

### Coding flank sequences effect V(D)J recombination efficiency, but not RSS selectivity

**Figure 3.**
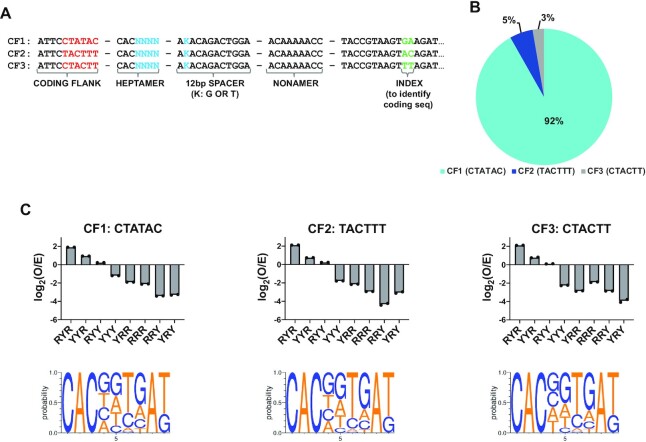
Coding flank effects on selectivity of 12-RSSs in V(D)J recombination. (**A**) The pSARP-cf12R4-7 input library contained three separate inserts that differed in coding flank sequence. The inserts are referred to as Coding Flank (CF)1, CF2 and CF3 with an AC, TTT or CTT sequence immediately flanking the RSS heptamer, respectively. The different coding flank sequences are in red text and index sequences for each insert are in green text. The degenerate portion of the RSS is shown in blue text with positions 4–7 of the heptamer fully randomized, and the second position of the spacer either a T or G (designated as K). (**B**) Pie chart depicting relative V(D)J recombination activity for each insert. The percentage values are the sum of the signal joint read counts for the respective insert divided by the total signal joint read counts for the pSARP-cf12R4-7 output library multiplied by 100. (**C**) Bar charts showing mean V(D)J recombination frequency of two technical replicates for indicated R/Y sequence motifs for the CF1 (left plot), CF2 (middle plot), and CF3 (right plot) sequences. Recombination frequencies are expressed as log_2_(O/E), as described in Figure 2 legend. Below each bar chart is the corresponding sequence logo. The probability of finding each base at positions 4–7 and bases G or T at position 9 is shown on the y-axis of the sequence logo. Positions 1–3 (CAC) and position 8 (A) are identical in all sequences.

Coding flank sequences immediately 5′ to the RSS heptamer impact V(D)J recombination activity (36–38). To test the effect of coding flank sequences on recombination efficiency and RSS selectivity, the pSARP-cf12R4-7 input library was synthesized. This library was generated using three separate coding flank sequences (Figure 3A; CF1–3 sequences listed in Supplementary Table S1) adjacent to a partially degenerate 12-RSS heptamer. The three coding flank inserts were combined in equal amounts prior to input library preparation (Supplementary Table S2). The pSARP-cf12R4–7 input library was co-transfected into HEK 293T cells with MBP-cRAG1 and Ch-cRAG2 expression vectors, and the remaining steps of the SARP-seq protocol followed (Figure 1A). After inversional V(D)J recombination, the coding flank is separated from the 12-RSS by 727 bp, and it can be removed by exonuclease activity prior to coding joint formation. Thus, each degenerate pMX construct contained a unique index upstream of the 12RSS nonamer to allow NGS read assignment of different coding flank sequences regardless of coding-end processing during VDJ recombination (Figure 3A).

The CTATAC coding flank (CF1) yielded the highest recombination activity at 92% of the total signal joint reads. The TACTTT (CF2) and CTACTT (CF3) coding flanks yielded 5% and 3% of total signal joint reads, respectively (Figure 3B and Supplementary Dataset S1), which is consistent with previous studies using similar coding flank sequences (36,37). Similar to the RSSspacer sequence, the coding flank affects the efficiency of V(D)J recombination. Our results suggest that a poly-pyrimidine coding flank is unfavorable for V(D)J recombination (Figure 3B). Although 12-RSSs flanked by the CF1, CF2 and CF3 sequences differed in overall recombination activity, each coding flank substrate largely maintained the R/Y selectivity profile observed in the pSARP-12R4-9 library (Figures 2C and 3C).

### R/Y heptamer selectivity profiles are similar for RSSs containing consensus or cryptic nonamers

**Figure 4.**
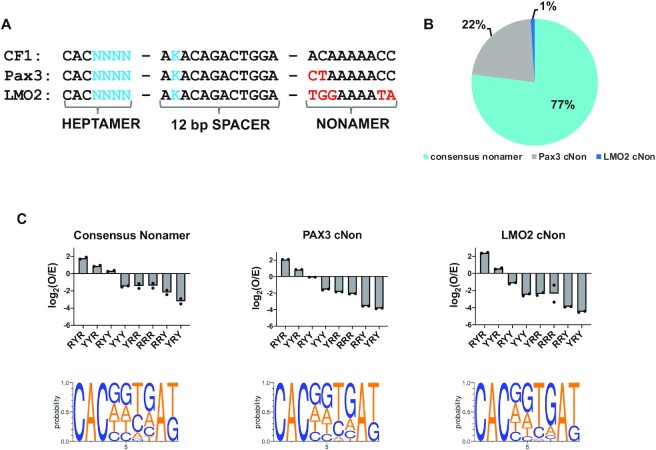
Effects of nonamer sequences on 12-RSS selectivity in V(D)J recombination. (**A**) The three different inserts in the pSARP-MAX-cNON input library included the CF1 insert (in Figure 3A), and inserts containing the cryptic nonamers present in known cRSSs found in the murine Pax3 and LMO2 gene loci. The heptamer, spacer and nonamer regions are denoted below the sequences. The degenerate sequences are in blue text as described in Figure 3 legend. Red text denotes sequence differences in the 12-RSS nonamer. (**B**) Pie chart depicting relative V(D)J recombination activity for each insert. The percentage values are the sum of the signal joint read counts for the respective insert divided by the total signal joint read counts for the pSARP-MAX-cNON output library multiplied by 100. The total signal joint read counts is derived from the sum of the signal joint reads for the Pax3 cNON, LMO2 cNON, and 15X the total read counts for the consensus nonamer insert. (**C**) Bar charts showing mean V(D)J recombination frequency of two technical replicates for indicated R/Y sequence motifs for the consensus nonamer (left plot), Pax3 (middle plot), and LMO2 (right plot) sequences. Recombination frequencies are expressed as log_2_(O/E), as described in Figure 2 legend. Below each bar chart is the corresponding sequence logo. The probability of finding each base at positions 4–7 and bases G or T at position 9 is shown on the y-axis of the sequence logo. Positions 1–3 (CAC) and position 8 (A) are identical in all sequences.

The RSS nonamer is a major binding site for the RAG1/2 complex, as it is recognized by the RAG1 nonamer-binding domain (NBD) (39). More divergent nonamers in endogenous and cryptic RSSs generally lead to decreased V(D)J recombination activity. Here, we tested if known cryptic nonamer (c-non) sequences could affect the selectivity of 12-RSS heptamers. The 12-RSS consensus nonamer in the pMAX-INV substrate was replaced with PAX3 (CTAAAAACC) and LMO2 (TGGAAAATA) c-non sequences (40). The PAX3 and LMO2 c-non inserts were combined at a 1:1 concentration. The consensus nonamer CF1 insert was added as a spike-in control at a relative concentration of 1:15 to each of the c-non inserts (Figure 4A). The combined inserts were ligated into the pMAX-INV vector to generate the pSARP-MAX-cNON input library (Supplementary Table S2), and the input library co-transfected into HEK 293T cells with Ch-cRAG1 and Ch-cRAG2 expression vectors. The remaining steps of the SARP-seq protocol were performed as in Figure 1A.

The PAX3 and LMO2 cNON containing substrates constituted 22% and 1% of the total signal joint read counts after normalizing for input concentration of the CF1 insert (Figure 4B and Supplementary Dataset S1). Interestingly, the output results from the pSARP-MAX-cNON library showed a slight preference for purines at position 4, in contrast to the lack of specificity at position 4 observed for either the pSARP-12R-4–9 or pSARP-cf12R4-7 libraries (Figure 4C). Further, the CF1 insert in the pSARP-MAX-cNON input library showed a slight, but detectable decrease in specificity at positions 6 and 7, as compared to the same insert in the pSARP-cf12R4-7 library (Figure 3C versus Figure 4C). The pSARP-cf12R4-7 and pSARP-MAX-cNON libraries were constructed using pMX-INV or pMAX-INV vectors, respectively, resulting in different promoters upstream of the degenerate 12-RSS. It is possible the presence of the strong CMV promoter in the pMAX-INV vector (Supplementary Figure S1) provides more favorable conditions for V(D)J recombination with the CF1 RSSs. Conversely, the poorer LMO2 cryptic nonamer appeared to lead to greater constraints on DNA specificity within the heptamer region such that the canonical G at positions 5 and 7 were preferred (Figure 4C). Nonetheless, many divergent heptamer motifs were still recombined when coupled with the LMO2 cryptic nonamer, demonstrating the wide range of substrates that can be targeted by RAG1/2 (Supplementary Dataset S1). We cannot completely discount that different fusion tags (MBP versus mCherry) for core RAG1 resulted in the subtle increase in purine preference at heptamer position 4 for heptamers flanked by CF1 in the pSARP-cf12R4-7 versus pSARP-MAX-cNON libraries. However, we believe this is unlikely because the respective tags on cRAG1 are fused to the N-terminal side of the RAG1 NBD and positioned far from the RSS heptamer, as indicated in previous cryo-EM studies (41,42).

### RSS R/Y motif adopts dynamic structural features unique to YpR base-pair steps

**Figure 5.**
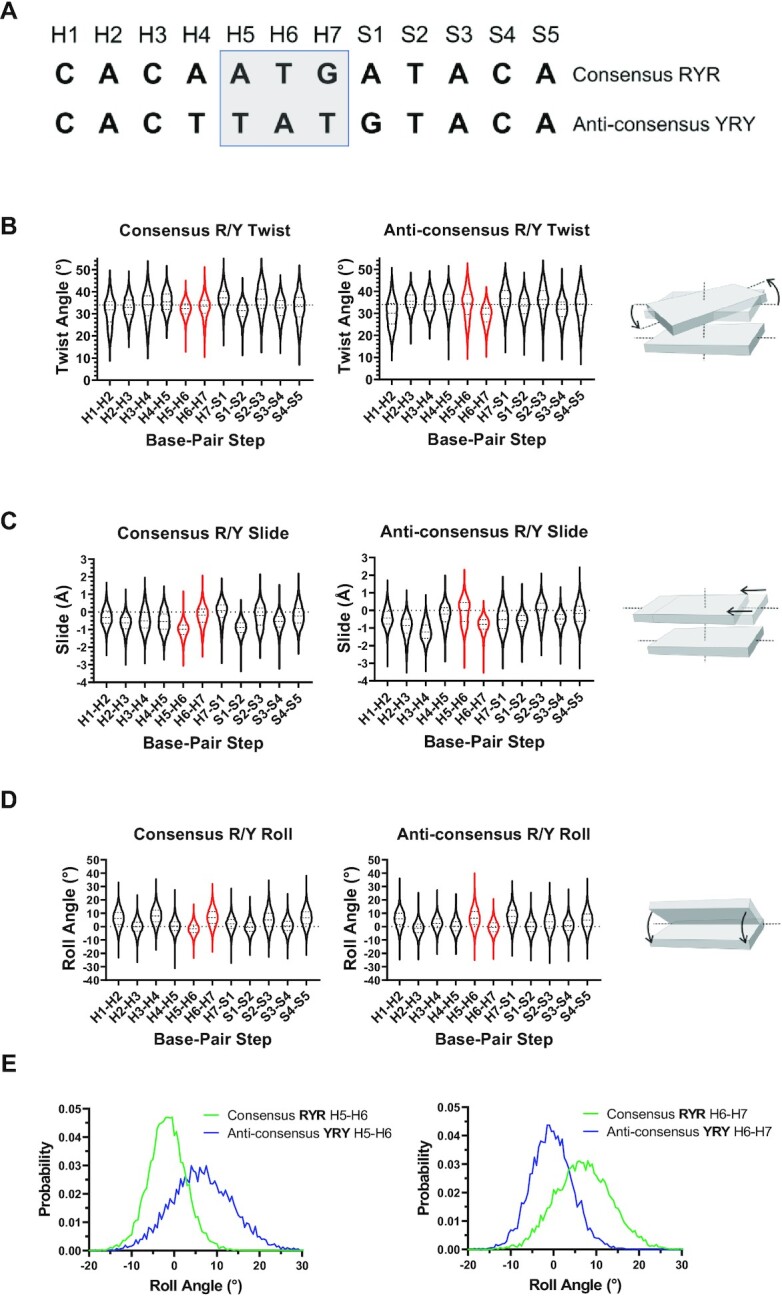
Molecular dynamics simulations of a consensus R/Y RSS (CACAATGAT) and an anti-consensus R/Y RSS (CACTTATGT). (**A**) Base position nomenclature for the 12-RSS heptamer and 5′ spacer region as used in subsequent panels. Violin plots depict probability distributions for (**B**) twist, (**C**) slide and (**D**) roll. Base-pair steps between heptamer positions 5–7 are colored red. Diagrams illustrating each base-pair step parameter is shown adjacent to the corresponding plots. (**E**) Roll angle probability distributions for base-pair steps H5–H6 (left) and H6–H7 (right). Plots for additional base-pair steps are shown in Supplementary Figure S8.

YpR steps can have unique base-pair step features that cause local distortions in a B-DNA helix (43,44). Recent cryo-EM and X-ray crystallographic data show the RSS heptamer untwists in the RAG1/2 active site prior to RSS cleavage (41,45,46). RAG-mediated cleavage therefore requires DNA flexibility, and RAG1/2 makes few base-specific heptamer contacts outside of the highly conserved CAC and dT at heptamer position 6 (41). While strong base-specific contacts with an intact, B-form RSS heptamer could enhance binding affinity and specificity, they could also hinder RAG1/2 catalysis by creating an energetic barrier to DNA structural transitions in the RAG1/2 active site. We hypothesized that the RSS heptamer motifs favored by RAG1/2 are structurally flexible, and RAG1/2 preference for R/Y motifs could be explained by dynamic structural features unique to RpY and YpR dinucleotide steps that accommodate heptamer untwisting in the RAG1/2 active site. To test this hypothesis, we performed *in silico* simulations of a highly active consensus R/Y RSS (CACAATGAT) and a poorly active anti-consensus R/Y RSS (CACTTATGT) (Figure 5).

The R/Y consensus RSS adopted unique twist distributions compared to the anti-consensus RSS (Figure 5B and Supplementary Figure S7). The first CpA base-pair step (H1–H2 in Figure 5A) adopted twist-angle probability distributions that were asymmetric for both the consensus and anti-consensus RSSs, a feature previously characterized in YpR steps (44). Other YpR steps in the simulated RSSs showed asymmetric twist-angle distributions characterized by an abundance of highly untwisted populations, including H3-H4 in the consensus RSS and H5-H6 in the anti-consensus RSS (Supplementary Figure S7). An exception to this was the TpG step (H6–H7) in the simulated R/Y consensus RSS heptamer, which did not have an asymmetric twist-angle distribution (Figure 5B, Supplementary Figure S7). We also measured base-pair slide and roll angle distributions for RSS base-pair steps (Figure 5C–E, Supplementary Figures S8 and S9). Consensus and anti-consensus RSS heptamers have markedly different slide and roll angle distributions for base-pair steps at, and proximal to, heptamer positions 5–7. The starkest contrast in roll and slide distributions are the base pair steps at H5–H6 and H6–H7 (Supplementary Figures S8 and S9). In both consensus and anti-consensus RSSs, the YpR step (H6–H7 for consensus RSS and H5–H6 for anti-consensus RSS) has a large roll angle and little-to-no slide (Supplementary Figure S11C), features that can induce DNA bending and major groove compression (47). The YpR step in the anti-consensus RSS is offset by one base position, which may incorrectly orient DNA structural distortions, such as unwinding, needed for RAG-mediated cleavage (41,46). Because RAG1/2 makes minor groove contacts proximal to H4–H6, we also measured RSS minor groove widths (Supplementary Figure S10). The consensus RSS heptamer had a wider range of minor groove widths, particularly at H2-H3, demonstrating its flexibility. Conversely, minor groove widths in this region of the anti-consensus RSS heptamer were more tightly distributed about smaller mean values, indicating a more rigid and narrow minor groove (Supplementary Figure S10). Minor groove flexibility may accommodate RAG1 interactions with the minor groove as well as helical distortions needed for heptamer unwinding (Supplementary Movie S1) (41,46). Because heptamer unwinding during RAG-mediated cleavage is caused by a combination of untwisting and helical distortions, we also measured minor groove widths when the first base-pair step (CpA) of the RSS heptamer is untwisted (H1–H2 twist angle < 17°) to characterize twist-coupled minor groove flexibility (Supplementary Figure S11A, B). Interestingly, the anti-consensus minor groove appears even more rigid and narrow when H1–H2 is untwisted, whereas the consensus RSS heptamer minor groove remains flexible. This suggests that the consensus R/Y motif better supports groove and base-pair step parameter flexibility than the anti-consensus R/Y motif, including twist-coupled minor groove deformations.

### Comparative analysis of SARP-seq results to RAG1/2 activity on endogenous RSSs

**Figure 6.**
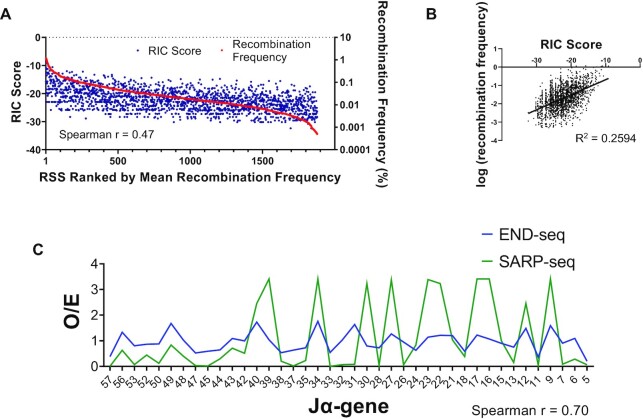
Endogenous RSS information content (RIC) scores and endogenous *Tcra* J-gene recombination compared with SARP-seq recombination. (**A**) Calculated RIC scores (blue dots, left y-axis) for each RSS analyzed in SARP-seq. Red line indicates mean RSS recombination frequency for each RSS characterized by SARP-seq (right y-axis, *n* = 3), and RSSs were ranked along the x-axis with most efficacious RSSs occupying higher ranks and less efficacious RSSs occupying lower ranks. (**B**) Log-transformed mean recombination frequencies for each RSS characterized by SARP-seq (*n* = 3) were plotted against RIC score. Black trend-line was generated using a linear regression model of log-transformed RSS recombination frequency expressed as a function of RIC score. (**C**) Mean normalized SARP-seq count frequencies and normalized END-seq count frequencies expressed as *O/E* where *O* is the observed frequency and *E* is the expected frequency if V(D)J recombination is completely random and nonspecific. END-seq expected frequency *E* was calculated by dividing the total END-seq counts for each RSS included in the analysis by the total number of unique RSSs being counted. Chromosomal position-specific effects were accounted for in the END-seq data by analyzing RAG cleavage efficiency of each J-gene relative to the cleavage efficiency of two flanking J-genes on either side (quantifications are provided in *Supplementary Dataset S2*).

Statistical models for RSS recombinogenic potential were previously developed (40,48), providing a scoring system for RSS information content (RIC). We calculated RIC scores for all RSSs observed in SARP-seq to compare RIC score to RSS efficacy determined by SARP-seq (Figure 6A and B). RIC scores were positively correlated with RSS efficacy (Spearman *r* = 0.47) (Figure 6A and B). Despite this, RIC was only somewhat predictive of RSS efficacy (Figure 6A and B). RIC scores were derived from conservation of endogenous RSSs, as opposed to relative RAG activity at each RSS. This likely accounts for the relatively poor correlation between RIC scores and SARP-seq results (Figure 6A and B), indicating that the importance of positions 4–9 in the 12-RSS for determining probability of RAG-mediated cleavage has been vastly underestimated.

RSS quality has been challenging to predict based on DNA sequence, since endogenous RSSs are embedded in a range of chromatin environments that can affect selectivity of individual RSSs by RAG1/2 (49,50). Examples of sequence-independent effects on RSS utilization include nucleosome density and positioning relative to the RSSs (51); the presence of certain histone marks, such as H3K4me3, that serve as a chromatin docking site for full length RAG2 (52); and 3-dimensional chromatin organization that can impact RAG1/2 access to RSSs (53). Here, we tested how the SARP-seq results correlated with RSS cleavage at an endogenous AgR locus. Specifically, we compared our SARP-seq results to the relative frequency of RAG-mediated cleavage across the murine *Tcra* J_α_ region, which was previously reported using the END-seq method (14). In this 64 kb region, there are 48 12-RSSs with CAC-containing heptamers (Figure 6C and Supplementary Dataset S2). Further, the *Tcra* J_α_-associated RSSs show considerable sequence variability through the heptamer and adjoining spacer region, providing an excellent test set to compare against the SARP-seq results. Previous studies have shown that the *Tcra* V_α_-to-J_α_ rearrangement is highly processive with attempts to join proximal V_α_ to the most 5′ J_α_ segments occurring prior to rearrangement of more distal 3′ J_α_ gene segments (54,55). These findings suggest RSS quality, based on DNA sequence, would not significantly factor into gene segment utilization. Nevertheless, we determined that there is a statistically significant correlation (Spearman correlation = 0.70, *P* < 0.0001) between RSS utilization for the END-seq versus SARP-seq results (Figure 6C and Supplementary Dataset S2). This is striking, considering that only the heptamer and the first 2 bp of the spacer can be exactly matched. RSS quality was generally better for more distal RSSs and poorer for more proximal RSSs (Figure 6C and Supplementary Dataset S2). The proximal RSSs would be within or near RAG-enriched recombination centers (54,55). Despite higher quality RSSs, more distal 12/23-RSS pairs were less frequently utilized during V(D)J recombination *in vivo* (54,55). These data suggest sequence-based RSS quality can help compensate for less frequent encounters of distal 12/23-RSS pairs. In summary, by comparing SARP-seq results from extrachromosomal substrates lacking specific chromatin modifications to chromosomal V(D)J recombination sequence specificity *in vivo*, it may be possible to discern *in vivo* RAG1/2 targets driven primarily by DNA sequence specificity from targets that are recombined due to epigenetic RAG1/2 effectors.

## DISCUSSION

Our development and implementation of SARP-seq, a high throughput V(D)J recombination assay, revealed the DNA sequence preference by the V(D)J recombination machinery for every functional 12-RSS heptamer sequence containing a ‘CAC’. These results provided a hierarchical ranking of RSS heptamers targeted by RAG1/2, showing that a broad range of heptamer sequences can be utilized in V(D)J recombination. The effect of the first two positions of the 12-RSS spacer on V(D)J recombination activity was also assayed, revealing heptamer sequence preferences for all 16 dinucleotide combinations. The present study also comprehensively assayed heptamer sequence preference in the context of four different coding flank sequences, three different nonamer sequences, and two different plasmid backbones. These different experimental contexts appeared to subtly influence RAG1/2 specificity; however, RAG1/2 R/Y motif preference was largely maintained. The YpR motif at heptamer position 6–7 was most favorable and the inverse RpY motif was least favorable in every experimental condition, consistent with our hypothesis that the YpR step may act as a flexible hinge to facilitate RAG activity. These results argue in favor of a more inclusive consensus RSS motif defined in terms of purine/pyrimidine content, as opposed to the strict base specificity implied by the canonical consensus CACAGTG heptamer motif.

SARP-seq experiments in the pMX backbone showed no specificity for adenine at heptamer position 4. This lack of specificity is consistent with recent structures of RAG:RSS complexes that showed little to no contact between RAG1/2 and heptamer position 4 (41). While it is possible that the non-core regions of RAG1/2, which are absent in the constructs used here, could affect preference at heptamer position 4, no previous studies have shown a role for the non-core regions in sequence-specific DNA interactions. Further, cryo-EM and X-ray crystallographic studies that included non-core RAG1/2 domains showed no resolvable cryo-EM/electron density for non-core RAG1/2 but extensive RAG-DNA contacts between the RSS and core RAG1/2 domains (41,45,46,56). Interestingly, SARP-seq experiments in the pMAX backbone had a slight bias towards purines at heptamer position 4, suggesting that RSS selectivity at heptamer position 4 is subtly influenced by the surrounding sequence context. The 12RSS in the pMAX backbone is flanked by a strong CMV promoter, while the pMX backbone contains retroviral LTRs without a strong internal promoter. Transcription-coupled epigenetic effects, such as DNA methylation, DNA supercoiling, or histone modifications may be subtly influencing RAG1/2 DNA sequence specificity, but given the small effect size in our study, future studies are needed to determine whether purine preference at heptamer position 4 is indeed dependent on transcription-coupled modifications. We also note that previous studies on RAG1/2 have found a strong preference for adenine at heptamer position 4 (14,48). However, these studies analyzed RAG1/2 specificity in a genomic context without isolating DNA sequence as an independent variable. Therefore, sequence motif preference could be influenced by myriad confounding variables including DNA accessibility, evolutionary biases in sequence motif abundance, topological associations, variable transcription coupled modifications (including H3K4 methylation activating RAG1/2 activity), etc. END-seq, for example, identified an abundance of RAG-mediated breakpoints at (CA)·(TG)-repeats (14), This could increase the apparent efficacy of ‘CACA’ heptamer motifs, but it might not reflect a real preference for ‘A’ at heptamer position 4. Future SARP-seq studies could be performed with non-consensus 23RSSs in a pre-B-cell line to more definitively determine if B-cell specific factors, ‘Beyond 12/23’ pairing rules, confounding variables, or unidentified V(D)J recombination factors are responsible for the previously observed preference for adenine at heptamer position 4.

A surprisingly large variety of sequences spanning heptamer positions 5–7 were targeted for V(D)J recombination in the SARP-seq experiments. Nevertheless, there was a clear preference for certain purine/pyrimidine (R/Y) motifs. R/Y RSS motifs containing a YpR step at heptamer positions 6–7 were the only enriched R/Y motifs found in precise signal joints (Figure 2C), highlighting its importance for RAG1/2 cleavage compared to other RSS heptamer features. Notably, YpR dinucleotides have low base-stacking energies and are among the most conformationally flexible base-pair steps (44,57,58). The deformability of YpR steps allows them to act as a flexible ‘hinge’ during protein:DNA interactions (58–60). Prior to nicking, RAG1/2 contacts T at heptamer position 6 through a wide minor groove, which may explain the preference for T over C (41,61). The RSS heptamer must unwind in the RAG1/2 active site to correctly position the scissile phosphate next to the catalytic residues (45). While the unwinding is mostly localized to the first 3 bases of the RSS heptamer, heptamer positions 4–7 are under-twisted, and the minor groove remains widened (41,46). The RSS heptamer must be flexible enough to accommodate these structural distortions prior to nicking. Because RAG1/2-mediated DNA cleavage is ATP-independent, the RSS heptamer must be able to unwind in the RAG1/2 active site without an external force driving the structural transition. These observations fit a model in which R/Y heptamer motif preference of RAG1/2 is a result of RAG1/2 selecting for DNA sequences that can accommodate the structural transitions necessary for RAG-mediated cleavage. Because SARP-seq analyzed the complete V(D)J recombination reaction, *in vitro* biochemical studies on RAG1/2 nicking specificity will help determine if R/Y specificity is due to sequence-mediated unwinding in the RAG1/2 active site.

The present SARP-seq study comprehensively assayed 12-RSS efficacy in the context of a consensus 23-RSS, similar to other studies on RAG1/2 specificity for the 12-RSS, but a non-consensus 23-RSS may affect coupled cleavage dictated by the ‘Beyond 12/23’ rule (62–65). To understand the basis of 12/23-RSS pairing, it would be optimal to analyze V(D)J recombination activity for a large set of 12/23-RSS pairs. The SARP-seq approach could be modified to incorporate partial degeneracy in both 12- and 23-RSSs to completely determine coupled cleavage efficacy for thousands of 12/23-RSS pairs. Although this would require a nontrivial modification to the input library preparation protocol, the resulting high-throughput analysis would yield insight into DNA sequence determinants required for optimal 12/23-RSS coupled cleavage.

SARP-seq is a powerful system for assaying DNA sequence specificity for the complete V(D)J recombination reaction, and analyzing DNA sequence in the absence of confounding variables present in genomic loci. This provides unique strengths and weaknesses in comparison to other established methods. For example, methods for assaying RAG1/2 specificity in a genomic context, such as END-seq, VDJ-seq, or ChIP-seq, have the advantage of assaying RAG binding, cleavage, or recombination of chromosomal substrates, but they fail to isolate DNA sequence as an independent variable (14,66,67). *In vitro* biochemical assays can separate specificity for binding, nicking, and cleavage, but such methods are further removed from a physiologically relevant *in vivo* context and ignore DNA repair. SARP-seq is a powerful tool that isolates DNA sequence as an independent variable for calculating relative efficacy of variant RSSs in the context of a cell, unlike any other method to date. However, we note that future *in vitro* biochemical studies performed on sequence motifs identified by SARP-seq would help identify which specific steps are affected by non-consensus RSSs, including the CACNRYR and CACNYRY motifs. Further, we cannot rule out that some sequences are more or less efficiently handed off to NHEJ machinery (68). While SARP-seq analysis of precise signal joints is highly relevant to the canonical V(D)J recombination reaction, it may underestimate cleavage of non-consensus RSSs that are less efficiently joined to the opposite signal end (68). Translocations proximal to non-consensus RSS-like sequences are frequently observed in lymphoid cancers expressing RAG1/2, but breakpoint junctions are not always precisely joined (18,69). Some imprecise signal joints contained small insertions or deletions, but non-NHEJ mediated end repair by homologous recombination or microhomology mediated end joining would not likely yield an identifiable signal junction. Building on the SARP-seq method to also include imprecise signal joints and alternative end-joining events will elucidate whether some non-consensus RSSs are more likely to be utilized in these pathways. Nevertheless, SARP-seq currently provides a useful reference for identifying cryptic RSSs that do not match the canonical CACAGTG heptamer motif.

Previous structural studies of RAG1/2 complexed with DNA revealed the structural mechanism by which RAG1/2 cleaves DNA (2), but there is no published structure of RAG1/2 in complex with a non-consensus RSS. Our results are consistent with many prior observations of the RAG:RSS complex, including the absence of base-specific contacts at heptamer position 4, the requirement for DNA deformability, the presence of a base-specific contact at heptamer position 6, and opening of the minor groove (41,45,46). However, one can only speculate on how the RAG1/2 active site would accommodate a non-consensus RSS. Current electron density maps and cryo-EM density maps are poorly resolved around the DNA bases, leading to conflicting reports on their orientation in the RAG1/2 active site (41,46). The present study showed that a consensus CACNRYR heptamer motif was conformationally flexible, and an anti-consensus CACNYRY motif was conformationally inflexible. Some sequences may also inhibit RAG-mediated cleavage by hindering these structural transitions through specific side-chain interactions. Because SARP-seq assays RSS efficacy for completing VDJ recombination, including end-joining, future biochemical studies are needed to determine whether some RSS motifs inhibit specific steps in the VDJ recombination reaction. Furthermore, solving structures of RAG1/2 in complex with non-consensus RSSs would provide insight into how the RAG1/2 active site accommodates divergent sequences.

The quality of RSSs may have evolved to modulate RAG1/2 activity at certain locations within AgR loci. In addition to the poor quality 5′ J_α_ RSSs in the mammalian *Tcra* locus (Figure 6C), the poor quality of V_β_ RSSs was proposed to reinforce allelic exclusion of *Tcrb* by limiting RAG1/2 activity (20,21). In a previous study, replacing a poor quality V_β_ RSS with a consensus RSS led to a dramatic increase in utilization of the adjacent V_β_ gene, as well as increased allelic inclusion, demonstrating the effect of RSS quality on V(D)J recombination efficiency in the context of an AgR loci (20,21). Still, it has been challenging to discern the contribution of RSS quality to RAG1/2 activity in varying chromatin environments. RAG-RSS interactions in AgR loci are also proposed to be driven by RAG1/2 scanning through cohesin-mediated DNA loops (70). Regardless of whether RAG recognizes RSSs through linear DNA scanning or three-dimensional diffusion, RSS quality could enhance or repress RAG-mediated cleavage by accommodating or resisting heptamer unwinding prior to nicking and hairpin formation. The R/Y motif preference in the heptamer determined by the SARP-seq results, along with evidence of heptamer base unwinding in RAG-RSS high resolution structures, are consistent with the importance of this region of the RSS in affecting the rates for nicking and hairpin formation.

The immense sequence variability in the 12-RSS and 23-RSS, along with 12/23-RSS pairing preferences, has been a formidable barrier to our understanding of DNA sequence specificity in V(D)J recombination. In this study, our comprehensive characterization of V(D)J recombinase activity on RSS heptamer sequences revealed preference for conformationally flexible R/Y motifs and even stronger selection against RSS heptamers with an RpY step at heptamer positions 6–7. Given the considerable sequence divergence of RSSs at some AgR loci, RAG1/2 specificity by exclusion may facilitate full AgR repertoire realization without compromising genomic stability. Future high-throughput experiments focusing on the RSS nonamer-spacer region and 12/23-RSS pairing will be needed to identify additional DNA sequence determinants governing V(D)J recombination and the production of antigen receptor repertoires.

## DATA AVAILABILITY

Next-generation sequencing data used in this study were deposited in NCBI Sequence Read Archive under BioProject Accession PRJNA786969. Galaxy scripts are included as supplementary data and available at the following link: https://github.com/RodgersLab/SARP-seq.git.

## Supplementary Material

gkac1038_Supplemental_Files

## Notes

Present address: Jennifer N. Byrum, Department of Microbiology and Immunology, University of Oklahoma Health Sciences Center, Oklahoma City, OK 73104, USA.

Present address: Karla K. Rodgers, Department of Microbiology and Immunology, University of Oklahoma Health Sciences Center, Oklahoma City, OK 73104, USA.
