# Supplementary material for: An updated definition of V(D)J recombination signal sequences revealed by high-throughput recombination assays
Source: Nucleic Acids Res. 2022 Nov 12;50(20):11696–711. doi: 10.1093/nar/gkac1038 (PMC9723617; doi:10.1093/nar/gkac1038)
Supplement: gkac1038_Supplemental_Files [file gkac1038_supplemental_files.zip › supplementary_data_NAR_revision2-wh-v5_10-18-22.docx]

**An Updated Definition of V(D)J Recombination Signal Sequences Revealed by High-Throughput Recombination Assays**

Walker Hoolehan, Justin C. Harris, Jennifer N. Byrum, Destiny A. Simpson, and Karla K. Rodgers

**Supplementary** **Data File**

**Figure S1.** Plasmid maps for the SARP-seq parent vectors. **(A)** The pMX-INV retroviral vector. eGFP refers to the gene encoding enhanced green fluorescent protein derived from *Aequorea victoria*. The internal ribosome entry site (IRES) facilitates bicistronic translation of GFP and hCD4 genes after recombination. **(B)** The pMAX-INV plasmid. ppluGFP2 is the gfp gene derived from *Pontellina plumata*. The locations of the 12-RSS and 23-RSS that flank the GFP gene are indicated in each plasmid. The GFP gene in each plasmid is in the forward orientation prior to V(D)J recombination. Following V(D)J recombination the GFP genes are inverted. The pSARP libraries were constructed by replacing the consensus 12-RSSs in each plasmid with the randomized sequences as described in the text.

**Figure S2.** SARP-seq control experiments **(A)** Selective PCR amplification of consensus RSS in pSARP-12R4-9 parent construct (pMX-INV) with and without RAG1/2 co-expression. Arrows indicate expected molecular weights for the input PCR product (higher arrow), which amplifies regardless of V(D)J recombination, and the signal joint PCR product which amplifies V(D)J recombination signal joints. **(B)** SARP-seq output library subjected to NGS. **(C)** Sanger sequencing electropherogram of partially degenerate 12-RSS that was subjected to the SARP-seq protocol *without* RAG1/2 expression **(D)** Sanger sequencing electropherogram of partially degenerate DNA sequence incorporated upstream of pSARP-12R4-9 and subjected to the SARP-seq protocol without RAG1/2 expression. The added degenerate DNA was used to further test whether the SARP-seq protocol skews base degeneracy.

**Figure S3.** Diagram of recombined extrachromosomal VDJ recombination substrates and PCR primers used to amplify VDJ recombination signal joints (SJ). *A)* Representative diagram of pSARP substrate in pMX-INV backbone. To prepare recombined output libraries from SARP-seq experiments utilizing the pMX backbone, primers “Nest FWD” and “Nest RVS” were used for the first round of PCR, followed by amplification with primer pair “P7 Primer” and “miSeq P5 Primer 1,” followed by the final round of PCR amplification with primers “P7 Primer” and “miSeq P5 Primer 2.” For the iSeq 1 library, primer “iSeq P5 Primer 1” was used in place of “miSeq P5 Primer 1” to generate the final output library. For semi-quantitative PCR assays in HEK293T cells expressing RAG1/2, primers “Nest FWD” and “Nest RVS” were used to amplify signal joints, and primers “Nest FWD” and “Input RVS” were used to amplify total input plasmid. *B)* Representative diagram of pSARP substrate in pMAX-INV backbone. To prepare output libraries, “MAX FWD” and “MAX RVS” were used for the initial round of PCR amplification. Subsequently, primers “P7 Primer” and “MAX P5 Primer 1” were used for PCR amplification. In the final round, primers “P7 Primer” and “miSeq P5 Primer 2” were used for PCR amplification. For semiquantitative PCR assays in the A70 pre-B-cell line, primer pair “MAX FWD” and “MAX RVS” was used to amplify signal joints, and primer pair “MAX FWD” and “MAX Input” was used to amplify total input plasmid.


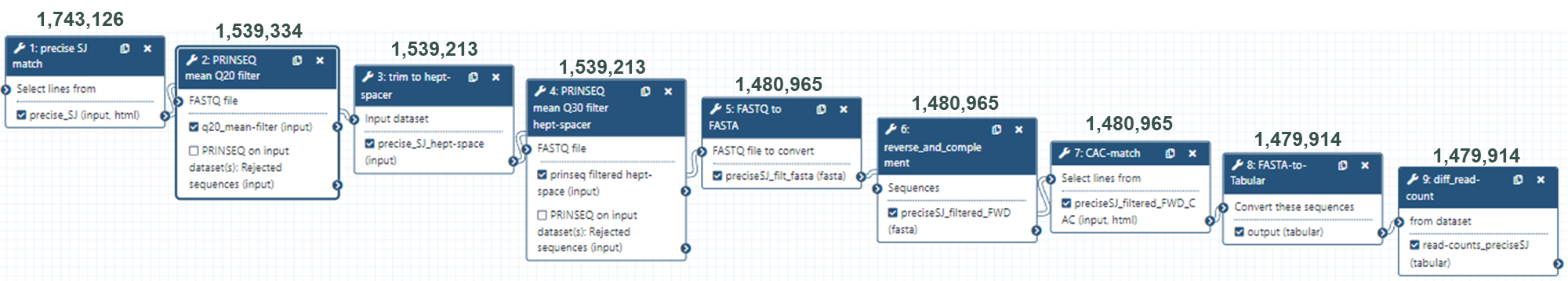


**Figure S4*A*.** Representative Galaxy workflow depicting bioinformatic pipeline for analysis of pSARP12R-4-9 output library (Galaxy script provided as supplementary file). The number of reads entering each step is depicted.


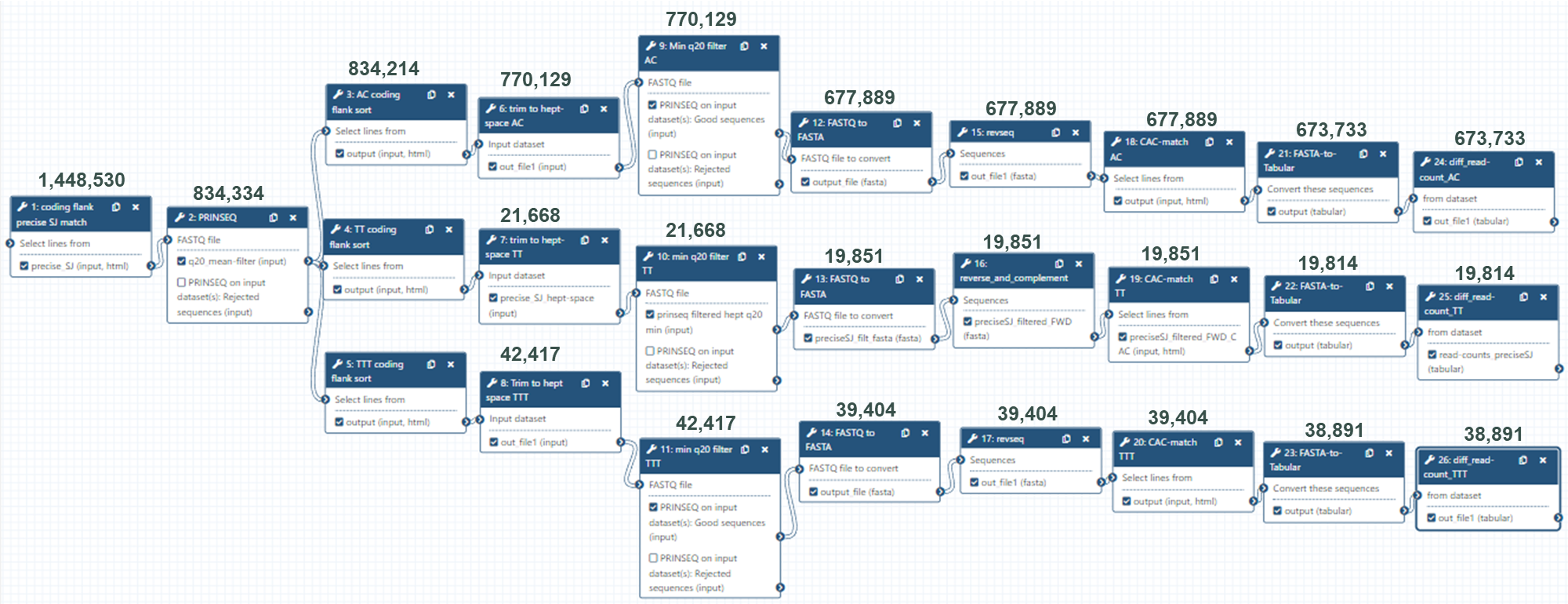


**Figure S4*B*.** Representative Galaxy workflow depicting bioinformatic pipeline for analysis of pSARP-cf12R4-7 output library (Galaxy script provided as supplementary file). The number of reads entering each step is depicted.


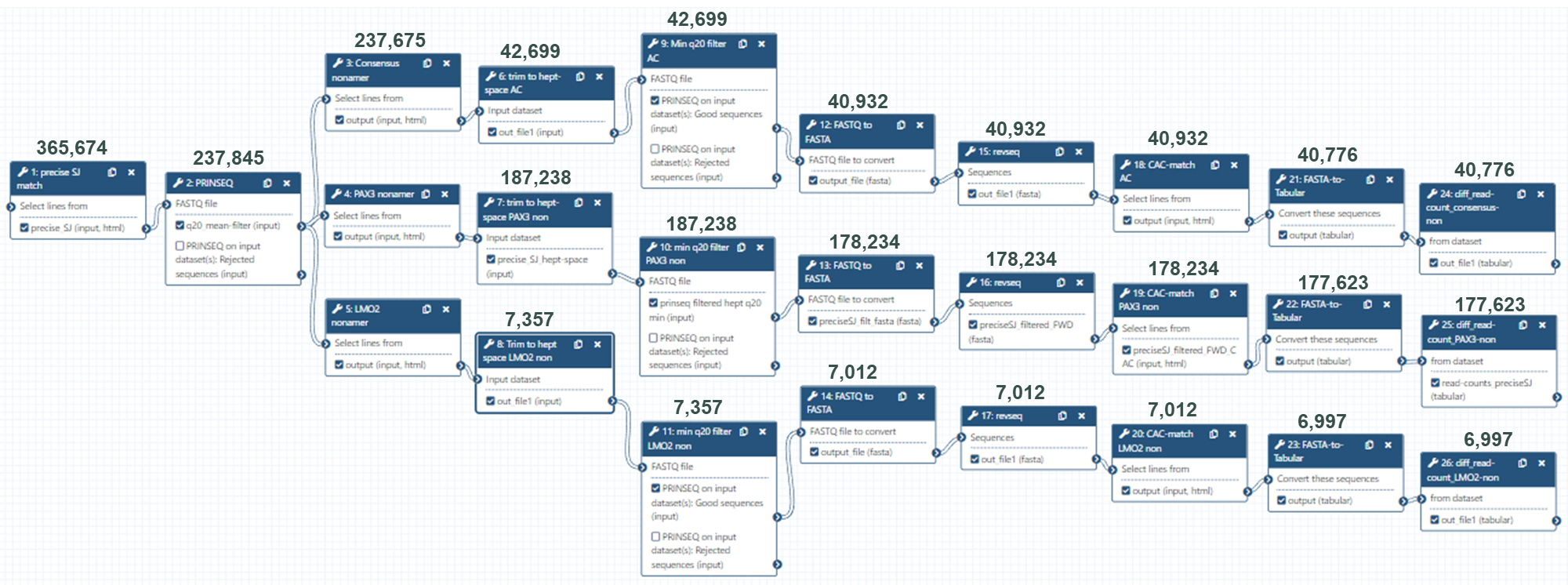


**Figure S4*C*.** Representative Galaxy workflow depicting bioinformatic pipeline for analysis of pSARP-MAX-cNON output library (Galaxy script provided as supplementary file). The number of reads entering each step is depicted.

Figure S5. RSS recombination frequency analysis (A) RSS recombination frequencies plotted with SEM of n = 3 replicates*.* (B) RSS recombination frequencies with minimum Q30 filtering plotted with SEM of n = 3 replicates. (C) RSS recombination frequencies with SEM expressed as log_2_(O/E) where *O* is the observed recombination frequency of an RSS and *E* is the expected recombination frequency of the same RSS if VDJ recombination was random and non-specific. (D) Recombination frequencies for respective RSS motifs where W = A/T and S = C/G. (E) Semi-quantitative PCR assay (left) and SARP-seq assay (right) comparing consensus RSS (CACAGTGAT) recombination to anti-consensus RSS recombination (CACGTACAT). (F) Semi-quantitative PCR assay performed in A70.2 pre-B cells expressing endogenous RAG1/2 (right) comparing consensus and anti-consensus RSS recombination (sequences as in panel E). Bar chart depicts mean and SEM for n = 3 replicates. Consensus and anti-consensus recombination was compared with a two-tailed Student’s t-test (*, *p* < 0.05; ***, *p* < 0.001)

Figure S6. Recombination frequencies for various RSS spacer sequences. (A) Mean recombination frequency for each RSS spacer sequence with SD for n = 3 replicates. *p*-values were calculated with an ordinary one-way ANOVA with Dunnett’s multiple comparisons test (**** denotes *p* < 0.0001, ns denotes *p* > 0.05). (B) Dot plots depicting mean recombination frequencies (n = 3) of each RSS heptamer sequence with corresponding spacer sequence on x-axis. (C) Mutual information ± SD shared between R/Y motifs for heptamer positions H5-H7 and all RSS spacer sequences (n = 3). Information content is expressed in nats and calculated using the equation *MI = H_h_* + *H_s_ ­*– *H_hs_* where *MI* is the mutual information shared between the spacer and heptamer R/Y motifs, *H*  is the entropy of RSS heptamer R/Y motifs (*H_h_*), spacer sequence motifs (*H_s_*), or joint heptamer-spacer sequence motifs (*H_hs_*) calculated using the equation *H = -∑Pln(P)* where *P* is the sequence motif probability (1). (D-F) Recombination frequency of each RSS heptamer R/Y motif with a (D) AT (E) TT or (F) TC spacer sequence. Bar charts depict mean and SD for n = 3 replicates.

**Figure S7.** Twist angle probability distribution of molecular dynamic simulation. The base-pair step is denoted at the top left of each plot for the consensus RSS (in green) and the anti-consensus RSS (in blue). Bases C4, C3, C2, and C1 are the coding flank sequence (5’-ACTG-3’). Bases H1-H7 are the heptamer sequence and S1-S5 are the flanking spacer sequence as shown in Figure 5A. RYR and YRY refer to the purine-pyrimidine motif at heptamer bases 5-7.

**Figure S8.** Roll angle probability distribution of molecular dynamic simulation. The base-pair step nomenclature is as described in Figure S7 legend.

**Figure S9.** Slide distance probability distribution of molecular dynamic simulation. The base-pair step nomenclature is as described in Figure S7 legend.

**Figure S10.** Minor groove width probability distribution of molecular dynamic simulation. The base-pair step nomenclature is as described in Figure S7 legend.


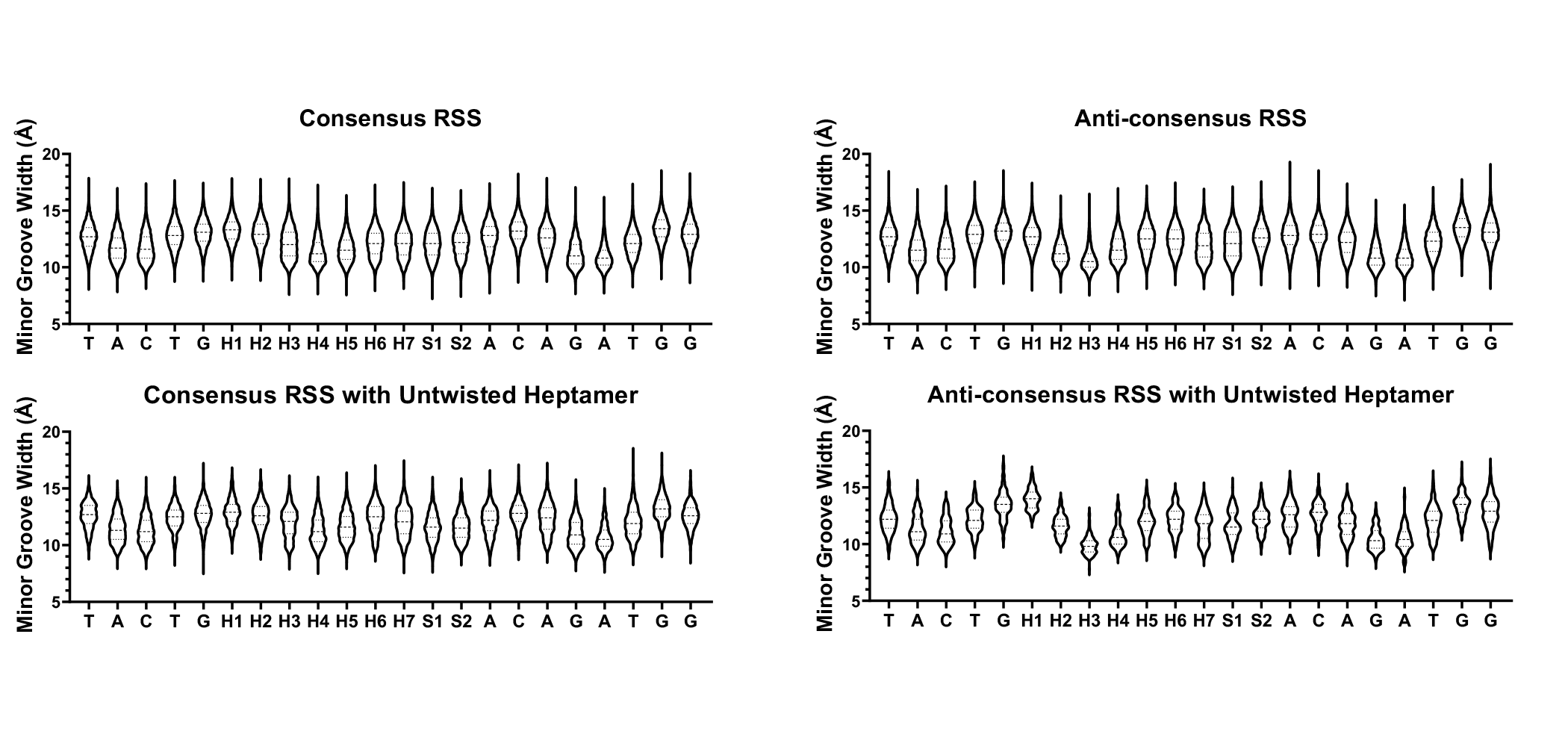


**B**

**A**

**C**

Figure S11. Molecular dynamics simulations of recombination signal sequences. (A-B) Top violin plots depict minor groove width measurements for every step of (A) consensus and (B) anti-consensus simulations. Bottom violin plots depict minor groove width measurements when base-pair step H1-H2 was untwisted (twist angle < 17°). Minor groove widths were measured as inter-phosphate distances using X3DNA “analyze.” (C) Tables of mean twist, roll, and slide values for base-pair steps contiguous to heptamer positions 5-7. The R/Y composition for each base-pair step is shown below each row of the columns, with YpR in boldface. Asymmetric probability distributions are highlighted yellow.

Table S1. SARP-seq primer list

| 12RSS4-9 | GTCTACGAACGAATTCCTACTGCACNNNNNNACAGACTGGAACAAAAACCAGATCGGAAGAGCGTCGTGTAGGGAAAGAGTGTAGATCTCGGTGGTCGCCGTATCATT |
| --- | --- |
| TT-CF-12RSS4-7 | GTC TAC GAA CGA ATT CCT ACT TCA CNNNNA KAC AGA CTG GAA CAA AAA CCT ACC GTA AGT TTA GAT CGG AAG  AGC GTC GTG TAG GGA AAG AGT GTA GAT CTC GGT GGT CGC CGT ATC ATT |
| AC-CF-12RSS4-7 | GTC TAC GAA CGA ATT CCT ATA CCA CNNNNA KAC AGA CTG GAA CAA AAA CCT ACC GTA AGT ACA GAT CGG AAG  AGC GTC GTG TAG GGA AAG AGT GTA GAT CTC GGT GGT CGC CGT ATC ATT |
| AC-CF-12RSS4-7 | GTC TAC GAA CGA ATT CTA CTT TCA CNNNNA KAC AGA CTG GAA CAA AAA CCT ACC GTA AGT GAA GAT CGG AAG  AGC GTC GTG TAG GGA AAG AGT GTA GAT CTC GGT GGT CGC CGT ATC ATT |
| Pax3Non12RSS4-7 | GTC TAC GAA CGA ATT CGGAACGTCTGCA CNNNNA KAC AGA CTG GA CTAAAAACC A GAT CGG AAG AGC GTC GTG TAG GGA AAG AGT GTA GAT CTC GGT GGT CGC CGT ATC ATT |
| Lmo2Non12RSS4-7 | GTC TAC GAA CGA ATT CGGAACGTCTGCA CNNNNA KAC AGA CTG GA TGGAAAATA A GAT CGG AAG AGC GTC GTG TAG GGA AAG AGT GTA GAT CTC GGT GGT CGC CGT ATC ATT |
| Duplexing primer | CAGTTCAGGTACGCGTAATGATACGGCGACCACCGAGATCT |
| Nest FWD | TCAAAGTAGACGGCATCGCAG |
| Nest RVS | TCGTCCTTGAAGAAGATGC |
| Input RVS | GAATGCTCGTCAAGAAGACAG |
| P7 primer | AATGATACGGCGACCACCGAGATCT |
| iSeq 1 P5 primer | CAAGCAGAAGACGGCATACGAGATTGGACGTAGCCTTCGGGCATGG |
| miSeq P5 primer 1 | GTGACTGGAGTTCAGACGTGTGCTCTTCCGATCTTGGACGTAGCCTTCGGGCATGG |
| miSeq P5 primer 2 | CAAGCAGAAGACGGCATACGAGATTAAGGCGAGTGACTGGAGTTCAGACGTGTGCTC |
| MAX FWD | CCA CTT TGC CTT TCT CTC CAC AGG |
| MAX RVS | TGC TCT TCA TCT TGT TGG TCA TGC GGC |
| MAX P5 primer 1 | GTG ACT GGA GTT CAG ACG TGT GCT CTT CCG ATC TTG CTC TTC ATC TTG TTG GTC ATG CGG C |
| MAX input | TAA CGT CTC GCC CTT TGG TCT CC |
| Vec FWD | CAG TGG AGT ACT ACC ACT GTG CAG CCT ACA ATT GAA TGC AGT GAA AAA AAT GCT TTA TTT GTG |
| Vec RVS | TGG AAC AAA AAC CTC GAC TTC GAA CGC GTC CTG TGG AGA GAA AGG CAA AGT GG |
| Insrt FWD | GCACAGTGGTAGTACTCCACTGTCTGGCTGTACAAAAACCGCC ACC ATG CCC GCC ATG AAG ATC GAG TGC |
| Insrt RVS | GTCGAGGTTTTTGTTCCAGTCTGTAGCACTGTGCAGACGTTCCgaattc GGC GAA GGC GAT GGG GGT CTT GAA GG |

**Table S2.** Overview of SARP-seq experiments for each input library

| **Library** | **pSARP12R-4-9** | **pSARP-cf12R4-7** | **pSARP-MAX-cNON** |
| --- | --- | --- | --- |
| **Parent plasmid** | pMX-INV | pMX-INV | pMAX-INV |
| **Insert** | Ns at positions 4-9 | CF1, CF2, and CF3 inserts (1:1:1)  Ns at 4-7; K at 9 | CF1 (in pMAX-INV):Pax3 c-non:LMO2 c-non (1:15:15)  Ns at 4-7; K at 9 |
| **Input library size**  **(# sequences)** | 4094^(1)^ | 1533^(2)^ | 1022 + 511 CF1 spike-in^(3)^ |
| **RAG constructs** | MBP-cRAG1 &  Ch-cRAG2 | MBP-cRAG1 &  Ch-cRAG2 | Ch-cRAG1 &  Ch-cRAG2 |
| **DNA purification protocol** | Modified Hirt procedure^(4)^ | Modified Hirt procedure | Total DNA extraction^(5)^ |
| **Illumina platform** | iSeq, miSeq | iSeq | iSeq |

(1) 4094 sequences expected at equal concentrations. 4096 (full degeneracy at 6 sites (4^6^)) - 2 (from digestion of 2 inserts with 6 bp sequences corresponding to MluI and EcoRI sites) = 4094.

(2) 1533 sequences at equal concentrations in total. 256 (full degeneracy at positions 4-7) X 2 (K nucleotide at position 9) – 1 (one sequence in library from position 3-8 corresponds to MluI site) = 511 for each coding flank insert.

(3) 511 sequences for LMO2 c-non and 511 sequences for Pax3 c-non at equal concentrations, and 511 sequences for the spike-in AC coding flank insert at 1/15^th^ concentration relative to the c-non inserts.

(4) Using the modified Hirt procedure (2), the cells were harvested by trypsinization, washed in PBS, and lysed by resuspension in digestion buffer (10mM Tris pH 7.5, 100mM NaCl, 25mM EDTA, 0.5% SDS, 0.1mg/mL proteinase K) overnight at 37°C with shaking. Cell lysate was equilibrated to 1 M NaCl by addition of 5 M NaCl solution, incubated overnight at 4°C, and the supernatant retained following 30 min 15,000g centrifugation at 4°C. The plasmid DNA in the supernatant was subsequently concentrated by ethanol precipitation and further purified with a Zymo DCC-25 kit. Ethanol precipitation was performed by adding 1/10 volume of 3M sodium acetate and 2 volumes of molecular biology grade 100% ethanol. Q5 High-Fidelity DNA Polymerase was used to amplify signal joints using the modified Hirt-recovered plasmid as template DNA.

(5) Total DNA extraction was performed using the modified Hirt procedure as described in footnote 4, except DNA from digested cells was recovered by direct ethanol precipitation of digested cells rather than salting out genomic DNA. Vent DNA Polymerase was used to amplify signal joints using the Total DNA extracted plasmid as template DNA.

Movie S1 (separate file). Movie showing RAG1 bound to minor groove of RSS heptamer. Movie was made using public data from a previous study (PDBID 6OEM) (3). White labels indicate heptamer positions H4 and H7. The RSS heptamer is colored red. RAG1 is represented as ribbon model with stick model of sidechains contacting the RSS minor groove.

Dataset S1 (separate file). Ranked SARP-seq read counts for reproducible SARP-seq reads depicted in Figure 2. RSS read count data for various coding-flank and cryptic nonamer sequences are shown in separate excel tabs. Read count data was used to generate all SARP-seq figures for this manuscript.

Dataset S2 (separate file). SARP-seq data compared to publicly available END-seq data (4) for *Tcra* J_α_-genes with 25 or more END-seq counts and an RSS heptamer sequence beginning with CAC. To account for position-specific effects on *Tcra* gene recombination, END-seq reads for each J_α_-gene were quantified relative to reads corresponding to 2 adjacent J_α_-genes. For example, J_α_-50 normalized count value was calculated from *O* observed reads divided by *E* which is the mean read count for J_α_-53, J_α_-52, J_α_-50, J_α_-49, and J_α_-48.

Galaxy Scripts. SARP-seq bioinformatic pipelines implemented as Galaxy workflows. “Galaxy-Workflow-SARP_v1_process.ga” was used to analyze SAMN23765598 FASTQ files. “Galaxy-Workflow-SARP_non-mix_process.ga” was used to analyze SAMN29354257 FASTQ files. “Galaxy-Workflow-SARP_cod-flank-mix_process.ga” was used to analyze SAMN29354256 FASTQ files.

Supplementary References

1. Cowell, L.G., Davila, M., Kepler, T.B. and Kelsoe, G. (2002) Identification and utilization of arbitrary correlations in models of recombination signal sequences. *Genome Biol*, **3**, Article Number: Research0072.0071.

2. Hirt, B. (1967) Selective extraction of polyoma DNA from infected mouse cell cultures. *J Mol Biol*, **26**, 365-369.

3. Chen, X., Cui, Y., Best, R.B., Wang, H., Zhou, Z.H., Yang, W. and Gellert, M. (2020) Cutting antiparallel DNA strands in a single active site. *Nat Struct Mol Biol*, **27**, 119-126.

4. Canela, A., Sridharan, S., Sciascia, N., Tubbs, A., Meltzer, P., Sleckman, B.P. and Nussenzweig, A. (2016) DNA Breaks and End Resection Measured Genome-wide by End Sequencing. *Mol Cell*, **63**, 898-911.
